# A Systematic Review of Recent Deep Learning Approaches for 3D Human Pose Estimation

**DOI:** 10.3390/jimaging9120275

**Published:** 2023-12-12

**Authors:** Amal El Kaid, Karim Baïna

**Affiliations:** Alqualsadi Research Team, Rabat IT Center, ENSIAS, Mohammed V University in Rabat, Rabat 10112, Morocco; karim.baina@ensias.um5.ac.ma

**Keywords:** 3D human pose estimation, systematic literature survey, deep-learning-based methods

## Abstract

Three-dimensional human pose estimation has made significant advancements through the integration of deep learning techniques. This survey provides a comprehensive review of recent 3D human pose estimation methods, with a focus on monocular images, videos, and multi-view cameras. Our approach stands out through a systematic literature review methodology, ensuring an up-to-date and meticulous overview. Unlike many existing surveys that categorize approaches based on learning paradigms, our survey offers a fresh perspective, delving deeper into the subject. For image-based approaches, we not only follow existing categorizations but also introduce and compare significant 2D models. Additionally, we provide a comparative analysis of these methods, enhancing the understanding of image-based pose estimation techniques. In the realm of video-based approaches, we categorize them based on the types of models used to capture inter-frame information. Furthermore, in the context of multi-person pose estimation, our survey uniquely differentiates between approaches focusing on relative poses and those addressing absolute poses. Our survey aims to serve as a pivotal resource for researchers, highlighting state-of-the-art deep learning strategies and identifying promising directions for future exploration in 3D human pose estimation.

## 1. Introduction

Human pose estimation (HPE) has become a challenging and prominent research area that has received great attention from the scientific community in the computer vision domain. By knowing the orientation and overall appearance of a person, we can understand human behavior and recognize activities within images or videos. This opens up numerous and diverse applications including in the gaming and animation industries, human–robot cooperation, bio-mechanical analysis in medicine, sports fields, gesture control, and autonomous driving [1,2,3,4]. The goal is to estimate joints (e.g., elbow, wrist) or part locations of a human associated with segments in a graphical form (skeleton representation) in order to characterize the 2D or 3D pose in the corresponding space.

In real-life scenarios, depth estimation becomes crucial for accurate pose estimation. While 2D poses can often be ambiguous, leading to similar appearances for different poses when viewed from different camera angles, 3D information helps resolve such ambiguities. Multiple approaches have been explored to deduce 3D human poses, including using depth images (RGB-D) that provide explicit 3D information, or leveraging multiple RGB images from different views to address occlusion challenges. However, the most commonly available input data are monocular RGB images, which pose challenges due to limited data availability, especially for uncontrolled or real-world images.

Early studies on 3D human body representation from image measurements date back to the 1970s [5,6,7,8]. These early works often modeled the human body as a collection of segments defined by overlapping spheres. In the 1980s, Ref. [9] inferred 3D articulations by sequentially determining the 3D coordinates of joints based on their 2D projections. Other approaches relied on engineering features, skeletal hypotheses, joint mobility assumptions [10,11], or image descriptors [10,12,13,14]. For example, Ref. [15] utilized shape contexts, Ref. [16] incorporated body part information, and [17] estimated 3D human positions using histograms of oriented gradients (HOGs) feature vectors. Generative models, such as the pictorial structure model (PSM), have also been widely used for pose estimation. Approaches like discretizing the pose space, as seen in [18], and employing it as a refinement step, as demonstrated in [19], showcase their versatility.

In recent years, deep learning paradigms have demonstrated remarkable success across various domains, including computer vision. Consequently, new methods for 3D human pose estimation increasingly rely on deep neural networks [20,21,22,23].

In the dynamic field of 3D human pose estimation, our study is distinguished by the use of a systematic literature review methodology to capture the latest advances in the field. In particular, we introduce a new taxonomy in addition to existing taxonomies that take into account two aspects: the type of input data and the number of people involved in the pose estimation. This innovative taxonomy encompasses other types and classifies multi-person 3D pose estimation into two categories: “root-relative” or “absolute”, depending on the type of estimation, a unique contribution not found in previous studies. In addition, we also organize video-based methods in a more comprehensible way according to their temporal information capture techniques, which improves the comprehensiveness of our study.

**Figure 1:**
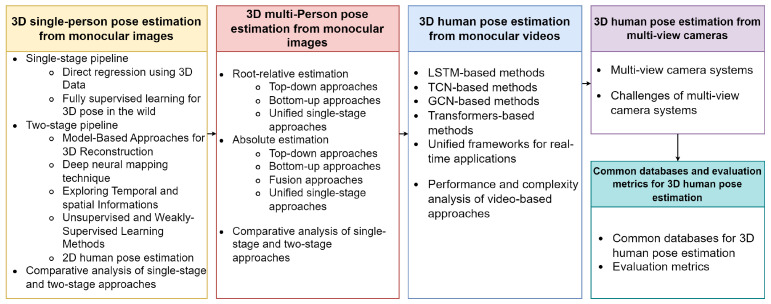
Roadmap of our survey’s structure.

In the subsequent sections, we provide a deep dive into the state-of-the-art techniques, introduce our unique taxonomy, and shed light on the prevailing challenges and potential future directions in 3D human pose estimation. A roadmap of our survey’s structure and content is illustrated in Figure 1.

## 2. Previous Surveys

The field of 3D human pose estimation has undergone substantial development, prompting numerous investigations over the years [24,25,26,27,28,29,30,31,32]. Many of these earlier surveys primarily focused on traditional methodologies based on handcrafted image descriptors.

The first survey on pose estimation, conducted by [24], provided a foundational overview of human motion capture approaches, which was later updated in [33]. Ref. [34] compared early approaches for 3D human pose estimation and activity recognition within the context of multi-view setups. Ref. [31] reviewed model-based approaches for recovering human pose in both 2D and 3D spaces, categorizing them based on appearance, viewpoint, spatial relationships, temporal consistency, and behavior. Ref. [26] explored single-view and multi-view human pose estimation, incorporating various input modalities such as images, videos, and depth data. In the subsequent year, Ref. [35] also published a survey on human pose estimation. Nevertheless, these surveys, conducted before 2016, predominantly focused on classical approaches employing handcrafted features and did not delve into deep learning techniques.

Later, Ref. [27] introduced methods for estimating human pose from monocular RGB images, including depth-based techniques. Ref. [25] discussed methods for estimating 3D human poses using RGB images and videos. Ref. [36] briefly summarized pose estimation methods as part of their exploration of deep learning applications.

Since 2019, further studies have emerged in this domain. Ref. [28] presented a comprehensive overview of 2D human pose estimation approaches rooted in deep learning, categorizing them based on single-person and multi-person estimation methods. Ref. [37] provided an overview of contemporary 2D pose estimation models, with a focus on architecture backbones, loss functions, and limitations. Multi-person pose estimation methods were the subject of investigation in [38,39], while [40] delved into deep models for 3D human pose estimation and offered comparisons of their strengths and weaknesses. Furthermore, Ref. [41] explored and evaluated six 3D reconstruction methods for monocular image sequences, with an emphasis on recovering 3D human pose from 2D joint locations. A comprehensive review up to 2020 was presented by [42], categorizing approaches into 2D and 3D scenarios, and further sub-categorizing 2D methods based on the number of individuals involved.

While several studies have been conducted on human pose estimation, there is currently no survey based on a systematic selection process. Efforts have been made to develop review methodologies, such as the selection of academic methods based on their relevance to different application fields and their performance on popular benchmarks [43,44]. However, given the increasing number of publications each year, there is a need for a systematic literature review in this area. Moreover, the previous surveys, based on our understanding, focus on single-person pose estimation methods in 3D or multi-person methods but only in 2D.

Therefore, the goal of this survey is to provide an up-to-date and credible overview of the most current methods and models for human pose estimation, with a focus on deep learning approaches. We also cover 3D multi-person poses from monocular images, which we categorize using two types of estimation: person-centric pose estimation and camera-centric pose estimation. Notably, these terms are commonly used in 3D estimation but have not been comprehensively addressed in previous surveys to the best of our knowledge. Within video-based methodologies, we organize the methods based on the specific models utilized to capture temporal information between successive frames, highlighting the key distinction among video-based methods. This survey constitutes a comprehensive study of the domain, encompassing all categories and types to assist researchers in determining the most suitable category for their specific scenarios.

The next section will describe the search strategy, research questions, and inclusion/exclusion criteria employed in this survey.

## 3. Survey Methodology

This section elucidates the methodology used in our systematic review of the most recent publications focusing on 3D human posture estimation based on deep learning from monocular cameras.

### 3.1. Research Questions

Our investigation is oriented by the following research questions (RQs):

- RQ1: What are the primary pipelines and taxonomies utilized in HPE?
- RQ2: What are the known approaches and associated challenges in different scenarios?
- RQ3: Which framework outperforms others in each case, and which techniques are required to mitigate these challenges?
- RQ4: What are the most widely used public databases and evaluation metrics in the field of 3D human posture estimation?
- RQ5: What are the current limitations and areas for future improvement in this field?

### 3.2. Search Strategy

The primary focus of this review is to provide an updated survey on 3D human pose estimation. In this context, we reviewed papers published from January 2021 to May 2023, adhering to the Preferred Reporting Items for Systematic Reviews and Meta-Analyses (PRISMA) [45] guidelines for paper selection to ensure relevant reporting.

The search process involved a manual exploration of specific publications within the field of human pose estimation, utilizing resources such as arXiv, Google Scholar, IEEE Xplore, ScienceDirect, Springer Link, and ACM digital libraries. The extraction of relevant papers was conducted on 10 June 2023.

The survey was further enriched with works published prior to 2021, where we selected the most renowned and innovative ideas that initiated a technology or a new philosophy in various scenarios to enhance our study. Additionally, a manual cross-reference search of relevant articles was conducted to identify other studies not previously discovered.

Further, we included a section on 2D pose estimation, with the criteria for inclusion being the most commonly used models in the field, and we selected a number of papers on multi-view pose estimation for comparative analysis.

### 3.3. Inclusion/Exclusion Criteria

In order to ensure the relevance and quality of the references included in this review, we applied a set of specific filtering criteria. These criteria were designed to help us identify and select the most pertinent and rigorous academic works in the field of 3D human pose estimation. The criteria encompassed the following:

- Searched and extracted conference proceedings and journal papers containing terms such as “3D human pose(s) estimation”, “3D multi-person pose(s) estimation”, “deep learning”, “monocular image(s)/video(s)”, or “single-view” in the title, abstract, or keywords.
- Included only online papers written in English and open access full texts.
- Considered only peer-reviewed articles, which were cross-verified in the Scopus database.
- Excluded direct duplicates and literature review papers to avoid redundancy.
- Prioritized the papers based on relevance and excluded those with weaker or less pertinent contributions.
- Included papers that provided novel methodologies, significant improvements, or substantial contributions to the field of 3D human pose estimation.
- Excluded papers that did not provide sufficient experimental results or lack rigorous methodological details.
- Included a select number of papers on multi-view pose estimation for comparative analysis, despite the main focus being on monocular pose estimation.
- For papers published prior to 2021, we focused on those that presented original ideas or marked significant improvements in the field.

### 3.4. Data Extraction, Analysis, and Synthesis

Initially, we screened the abstracts of the identified papers and retrieved the full texts of those deemed relevant. We thoroughly read the full texts to identify eligible articles, excluding those that deviated minimally from the original approaches or state-of-the-art counterparts.

During the data extraction process, we collected key information from the eligible articles, such as authors, publication year, methodologies, datasets used, evaluation metrics, and results. We organized the extracted data in a structured manner to facilitate comparison and analysis.

To ensure the quality and reliability of the included studies, we conducted a thorough quality assessment for each selected paper. This assessment considered factors such as the clarity of the methodology, experimental setup, and the rigor of the evaluation process. The aim of this assessment was to ensure that only high-quality and relevant studies were included in the review.

Based on the extracted data and the research questions, we synthesized the findings from the selected papers and organized them into coherent sections. This synthesis involved summarizing the main pipelines and taxonomies for human posture estimation, discussing the known approaches and challenges in different scenarios, and comparing the frameworks used in each case along with the techniques needed to address specific challenges. Through this data synthesis, we obtained a comprehensive overview of the current state of 3D human pose estimation using deep learning from monocular cameras.

In total, 62 papers were selected for this systematic review.These papers were chosen based on their relevance to the research questions and their contribution to the understanding of human posture estimation using deep learning approaches from monocular cameras.

## 4. Taxonomy of the Survey

Three-dimensional human pose estimation is a dynamic and diverse field of research in computer vision, enriched by a variety of specifications and properties that have stimulated the proposal of numerous taxonomy solutions. These approaches demonstrate considerable variation based on factors such as the type of input data, the number of individuals targeted, the learning paradigm employed, the specific pose estimation strategy implemented, and the coordinate system used for the results. While many prior surveys primarily classify methods according to their learning paradigms, our study structures these methodologies in a way that can better reflect the realistic use cases and practical considerations inherent in 3D human pose estimation.

Primarily, our categorization begins with the type of input data—monocular images or video, and multi-view images. Next, we consider the number of individuals involved in the case of monocular images. For video data, our emphasis shifts towards the type of network used for temporal information extraction across frames, disregarding the number of individuals (which is addressed similarly to the monocular image scenario). Lastly, in the multi-view context, we provide a brief description, primarily to draw a contrast with monocular setups, especially concerning the use of world or camera coordinates.

Thus, we organize the approaches reviewed in this survey into four main categories, each detailed in its corresponding section:

- **3D single-person pose estimation from monocular images**: In this section, we focus on approaches that aim at estimating the pose of a single person from monocular images. We further classify this into single-stage and two-stage pipelines, with the latter involving an intermediate 2D human pose estimation step.
- **3D multi-person pose estimation from monocular images**: This section broadens our review to methods designed to estimate the poses of multiple individuals from monocular images. We differentiate these based on whether they perform relative estimation or absolute pose estimation, with further subdivisions within absolute estimation into top-down, bottom-up, fusion, and unified single-stage approaches.
- **3D human pose estimation from monocular videos**: Transitioning from static images to video data, we review methods that are designed to estimate poses irrespective of the number of individuals. We categorize these methods based on the type of deep learning model they use, such as long short-term memory (LSTM), temporal convolutional networks (TCNs), graph convolutional networks (GCNs), transformers, or unified frameworks for real-time applications. A performance and complexity analysis of these methods is also included.
- **3D human pose estimation from multi-view cameras**: Lastly, we delve into methods that employ multi-view camera systems for human pose estimation, emphasizing how these methods exploit the additional depth information obtainable from multiple camera angles.

It is important to mention that many pose estimation methods use fully supervised learning. But, gathering large, labeled datasets with 3D pose details is often difficult and expensive. As a result, researchers are more and more interested in using self-supervised and weakly supervised techniques. These approaches aim to learn 3D pose estimation from single-view images, without needing exact 3D details during training. Instead, they might use consistency across multiple views, extract information from sequential input, or use other hints to infer the 3D pose from the data available.

In deep learning, although fully supervised learning is widely used, it faces difficulties because creating datasets with 3D annotations is complex. Manual annotation is particularly hard. Many 3D pose datasets are made using motion capture systems in indoor conditions, making them less useful in real-world settings. This means fully supervised methods have limited use, especially in practical or industrial situations.

To address these issues, researchers have explored other learning strategies, such as semi-supervised learning, weakly supervised learning, and unsupervised or self-supervised learning, as shown in Table 1. Semi-supervised methods often use only a small amount of annotated data, like 10% of 3D labels, showing that labeled training data are hard to come by. On the other hand, weakly supervised methods use existing or easily obtained hints for supervision, without needing exact 3D positions. These hints could include matched 2D ground truth data or camera parameters, among others. Lastly, unsupervised methods do away with the need for multi-view image data, 3D skeletons, correspondences between 2D and 3D points, or previously learned 3D priors during training. The Table 2 provides a detailed explanation and meaning of the technical terms and networks discussed in this paper.

## 5. Three-Dimensional Single-Person Pose Estimation from Monocular Images

Single-person pose estimation is employed to identify and pinpoint joint coordinates and human body parts in scenarios where the individual is the primary subject of the 2D image or video frame. The ultimate goal is to reconstruct the 3D pose of the person. This technique not only yields significant results on its own, but it also serves as a key component in top-down multi-person estimation methods, where it is applied to each human bounding box. Despite the potential challenges associated with single-person pose estimation, it provides superior accuracy and efficiency when analyzing the poses of individual persons.

The earliest approaches for 3D human pose estimation from single images [13,14,15,73,74,75] were generally based on discriminative methods and viewed the pose estimation as a regression or a classification problem. A mapping function is learned from a space of image features, that are either extracted directly as shape context [15,76], segmentation [14], silhouette [77], HOG [19,78], SIFT [73], or computed as body part information [16] to the pose in 3D space. This mapping must be well generalized to accurately estimate a 3D pose from a test image that has never been seen before. The outstanding results of deep learning methods in computer vision have made it a general trend to use deep nets to automatically learn the key information in images. Some papers rely on supervision learning to directly regress joint locations and predict the 3D pose from 2D monocular images without intermediate supervision [46,79,80,81,82]. In this case, models must be trained using a 3D pose annotated images dataset. For example, the Human3.6M [13] and HumanEva-I [83] datasets contain images captured in controlled environments using MOCAP systems. Thus, they prevent the models generalizing well to images in different environments, as in the wild. To address this issue, some approaches use both 2D datasets in the wild and 3D MOCAP datasets to guide and improve the training.

Monocular view is a critical aspect of 3D human pose estimation from monocular images. Unlike multi-view images or stereo images, which provide additional depth information, monocular images only provide a single 2D view of the scene. As a result, algorithms must use only the information contained in that single image to estimate the 3D pose of the human subject. This can be a challenging task, as the loss of depth information can make accurately estimating the pose more difficult. However, there are also advantages to using monocular images. For instance, monocular images are often easier to acquire, requiring only a single camera rather than multiple cameras. Additionally, they can be more widely used in real-world scenarios, as they do not require specialized equipment or setups. Therefore, while the lack of depth information in monocular images can pose a challenge, it also provides an opportunity for researchers to develop more sophisticated algorithms and techniques for accurately estimating the 3D pose of human subjects from single-camera images.

**Figure 2:**
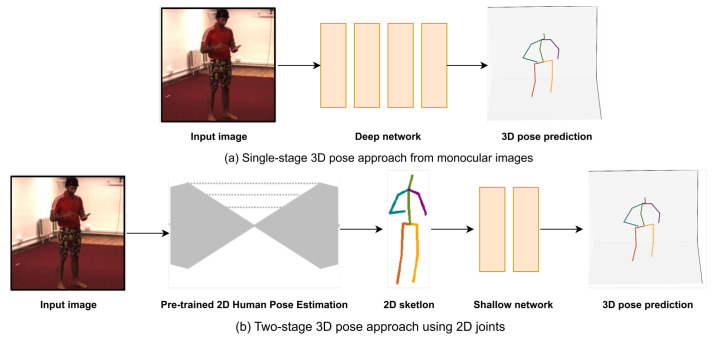
Techniques for 3D human pose estimation.

In 3D human pose estimation, techniques can generally be divided into two main categories: single-stage pipeline and two-stage pipeline, as presented in Figure 2. The single-stage pipeline consists of direct methods designed to derive 3D poses directly from input data, which may include images, videos, or depth maps. Conversely, the two-stage pipeline involves methods that typically employ 2D estimator networks at an intermediate stage. These categories are also applicable in multi-image contexts (multi-view or video), and some examples are cited in the subsequent section to provide a comprehensive overview without duplicating the content in the section dedicated to video analysis. However, the temporal information specific to videos will be elaborated upon in Section 7.

### 5.1. Single-Stage Pipeline

Single-stage pipeline based on direct regression approaches forms a significant category in 3D human pose estimation. The fundamental idea of this category is to directly predict the 3D coordinates of human joints from input data. Ref. [81] introduced an end-to-end regression architecture that achieved structured prediction by incorporating a pre-trained autoencoder at the top of traditional CNN networks, rather than directly regressing joint coordinates. With the autoencoder, they were able to learn a high-dimensional latent pose representation and account for joint dependencies. The same authors proposed a method in [82] to learn a regression model for 3D pose mapping from videos using CNNs. To ensure the validity of the predicted poses, Ref. [80] embedded a kinematic object model into a deep learning algorithm to regress joint angles of a skeleton. They defined a continuous and differentiable kinematic function based on bone lengths, bone connections, and a definition of joint rotations. This function was integrated into a neural network as a special layer, called a kinematic layer, to map the motion parameters to joints. Ref. [46] proposed another approach to reduce data variance, using a bones representation instead of joints in a structure-aware regression approach. They defined a compositional loss function that encoded long-range interactions between these bones based on the joint connection structure.

The rest of this section reviews recent advancements in this area up to 2021 that employ innovative techniques to improve the performance of regression-based pose estimation.

#### 5.1.1. Direct Regression Using Only 3D Data

Ref. [84] introduces an intuitive physics-based network (IPMAN) that regresses 3D human poses directly from 2D images. This work is distinctive in that it incorporates a physics-inspired loss function, which enforces physical plausibility in the predicted poses. However, it is worth noting that the idea of using physical constraints to enhance the plausibility of estimated poses is not novel. Shimada et al. [85] proposed a method for neural monocular 3D human motion capture that also includes physical awareness, attesting to the effectiveness of such an approach. Similarly, Huang et al. [86] proposed a method for capturing and inferring dense full-body human–scene contact, similar to the concepts presented in the IPMAN approach, which also focuses on body–ground contact and other physical interactions. Likewise, Shi et al. [87] presents a method that enforces skeleton consistency, using a body model to constrain the estimated poses. Further, this approach reconstructs 3D human motion from monocular video. These methods demonstrate the importance and effectiveness of incorporating physical constraints and constraints in the 3D human pose estimation field.

On the other hand, a recent study by Luvizon et al. [88] presented SSP-Net, a scalable convolutional neural network architecture specifically designed for real-time 3D human pose regression. SSP-Net addresses the challenges associated with varying input sizes and model complexities. Particularly, its pyramid structure enables multi-scale processing, capturing a wide range of details and contextual information. SSP-Net incorporates intermediate supervisions at different resolutions, refining pose predictions and improving accuracy. Furthermore, the sequential design of SSP-Net allows for iterative refinement of pose estimation, resulting in enhanced accuracy and real-time performance, enabling predictions at a frame rate of approximately 200 fps.

In their research, Kundu et al. [72] went beyond solely relying on supervised methods and explored the paradigm of self-supervised learning. They introduced MRP-Net, a model that not only predicts outputs but also estimates its own prediction uncertainty. This uncertainty-aware adaptation framework enhances the model’s performance in handling diverse domains within the self-supervised learning framework. By integrating self-supervised learning and quantifying prediction uncertainty, this research demonstrates the potential for more robust and adaptable 3D human pose estimation in challenging scenarios such as occlusion and truncation. Direct regression techniques in 3D human pose estimation extend beyond single-person or monocular images. They have been successfully applied to videos, as demonstrated in works such as Honari et al. [71] and Luvizon et al. [89]. These studies explore temporal information to improve pose estimation accuracy over time. Furthermore, direct regression approaches have been extended to handle multi-view scenarios, as shown in Zhang et al. [90]. These methods leverage information from multiple camera views to achieve more accurate 3D pose estimation. Additionally, direct regression techniques can be adapted to handle multi-person scenarios, as exemplified in the works of Sun et al. [91] and Wang et al. [92]. Detailed explanations of these articles are provided in the subsequent sections.

#### 5.1.2. Fully Supervised Learning for 3D Pose in the Wild

Convolutional neural networks (ConvNets) have shown impressive results in 3D pose estimation, particularly when trained on 3D data. They employ supervised learning to directly regress joint locations and predict the 3D pose from 2D monocular images without intermediate supervision. These models require training using a dataset of annotated 3D pose images, which are relatively rare, such as the Human3.6M and HumanEva-I datasets that contain images captured in controlled environments using MOCAP systems. However, these models often struggle to generalize to images captured in uncontrolled, or ‘in-the-wild’, scenarios. To tackle this issue, researchers have explored end-to-end approaches that utilize both 2D datasets from ‘in-the-wild’ scenarios and 3D MOCAP datasets to guide and enhance the training process. These approaches fall into two main categories.

##### Integrated Feature Sharing Models

The first category refers to training a single model that shares intermediate CNN features between 2D and 3D joint locations. For example, Park et al. [93] developed an algorithm that exploits image features and 2D pose estimation results as inputs, learning the relationship between 2D and 3D poses. In parallel, Pavlakos et al. [93] employed a fine-tuning strategy with 2D data to predict 3D joint locations, introducing a coarse-to-fine supervision learning scheme to improve initial estimates. In a significant contribution to this category, Mehta et al. [94] presented the first real-time method to capture the full global 3D skeletal pose of a human using a single RGB camera. Their method combines a new CNN-based pose regressor with kinematic skeleton fitting, regressing 2D and 3D joint positions jointly in real time. This approach has been shown to be applicable in real-time applications such as 3D character control and works well even in outdoor scenes, community videos, and with low-quality commodity RGB cameras. Tome et al. [95] further proposed a multi-stage CNN architecture, integrating a pre-trained layer based on a probabilistic 3D pose model into the convolutional pose machine (CPM). This approach lifts 2D landmark coordinates into 3D space, propagating the 3D skeletal structure information to the 2D convolutional layers. Similarly, Ghezelghieh et al. [96] predicted 2D and 3D poses by incorporating camera viewpoint information and 2D joint locations to achieve global joint configuration information. More recently, a method known as orthographic projection linear regression for single-image 3D human pose estimation was introduced in [97], tackling the small-angle problem in reprojection-based approaches and reducing overfitting risks in the depth dimension. Additionally, exemplar fine-tuning (EFT) for 3D human pose fitting [98] was also proposed to expand in-the-wild 3D pose collections without the need for specialized capture equipment. EFT fits a 3D parametric model to 2D keypoints, overcoming depth reconstruction ambiguity inherent in 2D inputs.

##### Combined 2D and 3D Data Learning Models

The second category comprises approaches that combine 2D and 3D data to learn a pose regressor. Zhao et al. [46] proposed a compositional pose model that jointly learns 2D and 3D poses, as well as action recognition, using an intermediate volumetric representation for 3D poses. In a similar vein, Luvison et al. [99] developed a multitask deep model that simultaneously learns 2D and 3D poses, along with action recognition, in an end-to-end trainable manner. Additionally, Li et al. [100] proposed a regression approach estimating 3D pose through a nearest neighbor form between images and poses. Their network learns a similarity score function between the feature embedding of the input image and the 3D pose, an essential step for pose estimation in an image. Adding to this category, Zhou et al. proposed a weakly supervised transfer learning method that uses a mix of 2D and 3D labels in a unified deep neural network [101]. This network has a two-stage cascaded structure, augmenting a state-of-the-art 2D pose estimation sub-network with a 3D depth regression sub-network. Their training is end-to-end and fully exploits the correlation between the 2D pose and depth estimation sub-tasks, allowing the 3D pose labels in controlled laboratory environments to be transferred to in-the-wild images. They also introduce a 3D geometric constraint to regularize the 3D pose prediction, which is effective in the absence of ground truth depth labels. Their method achieves competitive results on both 2D and 3D benchmarks.

### 5.2. Two-Stage Pipeline

Two-stage approaches to 3D human pose estimation employ a distinct two-step methodology. The process begins with the estimation of 2D joint locations within an image, which are then elevated or ‘lifted’ into the 3D space. This procedure effectively combines 2D pose estimation with 3D pose reconstruction. The 2D pose estimation can be achieved through heatmap-based or regression-based strategies, which will be explored in the subsequent subsection. Meanwhile, 3D pose reconstruction can be accomplished using techniques like triangulation or model-based optimization [102]. Contrary to the current approach of employing deep neural networks to learn the correlation between 2D and 3D poses, early works elevated 2D poses to 3D by identifying the most suitable 3D posture that corresponds to the 2D observations in a complete dictionary of 3D poses learned from large 3D pose databases using principal component analysis (PCA) or another dictionary learning method. Some methods, such as the one proposed by [103], minimize a loss on dictionary coefficients and joint speed, which is conditioned by the pose. This approach has been refined by [104,105] using a convex approach and an expectation maximization algorithm, respectively, to jointly estimate the coefficients of the sparse representation. Other methods, such as those proposed by [1,106], focus on matching the depth of the 2D poses using the k-nearest neighbor algorithm. In contrast, Ref. [107] proposes a method that minimizes the projection error under the constraint that the solution is close to the retrieved poses.

#### 5.2.1. Model-Based Approaches for 3D Reconstruction

In the literature, several papers have relied on model-based approaches for 3D reconstruction from 2D keypoints, as the approach used in [108], which treats pose estimation as a classification problem over a set of pose classes, with each image being assigned to the class with the highest score. This ensures a valid pose prediction but is limited to the existing classes. As a result, the pose obtained is essentially an approximation. The precision of the classification approaches increases with the number of classes, but this also complicates discrimination. In Ref. [103], a dual-stream CNN was employed to detect 2D joint landmarks using both original images and height maps, which encode depth information. Following the detection phase, they formulated an objective function to estimate the 3D pose. This function minimized a loss based on the coefficients of a 3D pose dictionary and pose-conditioned joint velocity, effectively transforming the 2D pose into a 3D pose.

A common methodology in two-stage approaches to 3D human pose estimation is to create a comprehensive basis of 3D poses. This facilitates the estimation phase. Zhou et al. [104,105] developed a shape dictionary by aligning all 3D poses in the training set using the Procrustes method. This approach succinctly summarizes the variability in training data and enables a sparse representation. They then proposed a convex approach to jointly estimate the coefficients of the sparse representation. Similarly, Chen et al. [106] and Gupta et al. [1,109] used a large library of 2D keypoints and their corresponding 3D representations to match the depth of the 2D poses using the k-nearest neighbor algorithm. Ramakrishna et al. [10] built a sparse representation of 3D human pose in an over-complete dictionary and proposed a projected matching pursuit algorithm to estimate the sparse model from only 2D projections. Contrasting these methods, Yasin et al. [107] and Simo-Serra et al. [110] focused on addressing 2D pose estimation errors. Simo-Serra et al. proposed a Bayesian framework that integrates a generative kinematic model and discriminative 2D part detectors based on histogram of oriented gradients (HOGs) to generate a set of 3D pose hypotheses. Yasin et al. combined two independent training sources using a dual-source approach. They retrieved the nearest 3D poses using the estimated 2D pose and reconstructed the final 3D pose by minimizing the projection error under the constraint that the solution is close to the retrieved poses. Another strategy integrates the generative 3D body shape model with the skinned multi-person linear (SMPL) model [111] to reconstruct 3D pose and shape. Bogo et al. [112] proposed a method called SMPLify, which first uses DeepCut [113] to generate 2D body joint locations. These locations are then fit with the SMPL model to predict 2D keypoints. The fitting is driven by an objective function that matches the projected 3D model keypoints and detected 2D keypoints. Tripathi et al. [61] proposed an unsupervised method that uses the 3D keypoints predicted by another network as pseudo ground truth in training. In a novel approach, Arnab et al. [98] proposed exemplar fine-tuning (EFT) to augment existing 2D datasets with high-quality 3D pose fits. EFT combines the re-projection accuracy of fitting methods like SMPLify with a 3D pose prior implicitly captured by a pre-trained 3D pose regressor network. This method results in high-quality 3D pseudo-annotations that improve downstream performance and are qualitatively preferable in an extensive human-based assessment. The authors also introduced new benchmarks for the study of real-world challenges such as occlusions, truncations, and rare body poses.

#### 5.2.2. Deep Neural Mapping Techniques

Deep neural mapping techniques, employing fully connected, convolutional, or recurrent networks, have revolutionized the field of 3D human pose estimation from 2D keypoints. These techniques leverage the power of deep learning models to effectively resolve complex, non-linear transformations. This represents a significant shift from example-based approaches, which rely on a dictionary or predefined base of 3D poses. Instead, deep neural mapping approaches learn the mapping directly from the data, enabling them to potentially capture a wider range of poses and more complex relationships between 2D and 3D poses. Moreno-Noguer [114] utilized a convolutional pose machine to detect 2D joint locations, and then inferred 3D poses through Euclidean distance matrix regressions, with the final 3D pose obtained using multidimensional scaling. This approach was further developed by Martinez et al. [115], who employed a multilayer perceptron to regress 3D joint locations from 2D keypoints, which were predicted by a stacked hourglass network. This demonstrated a similar application of deep neural mapping. Taking a different approach, Mehta et al. [116] used transfer learning to apply knowledge from 2D joint location to 3D pose estimation, showcasing the versatility of deep learning techniques in this domain. Building on these methods, VNect [94] integrated a CNN with a kinematic skeleton fitting to generate temporally stable full 3D skeletal poses, enabling real-time 3D pose estimation.

Moving forward, it is important to highlight the innovative approaches to 3D human pose estimation that have been proposed in recent studies up to 2021. Wu et al. introduced an improved mixture density network for 3D human pose estimation with ordinal ranking [117]. This method leverages mixture density networks (MDNs) to predict multiple 3D pose hypotheses, allowing the network to learn the Gaussian distribution of human body poses. Additionally, an ordinal matrix is introduced to select the correct pose estimation, highlighting the ability to handle ordinal ranking.

#### 5.2.3. Exploring Temporal and Spatial Information

Diving into diffusion-based approaches, Choi et al. introduced DiffuPose [49], which utilizes a graph convolutional network (GCN)-based architecture for lifting 2D keypoints to 3D. By treating the human skeleton as a graph, with joints as nodes, the lightweight GCN-based architecture captures topological information between joints. The diffusion process then refines the 3D pose estimation by propagating information across the graph, enhancing the overall accuracy. Another approach that leverages the power of graph convolutional networks (GCNs) is presented by Zeng et al. with their work on learning skeletal graph neural networks for hard 3D pose estimation [118]. Their method introduces a hop-aware hierarchical channel-squeezing fusion layer, a sophisticated technique that takes into account the distance between nodes (hop-aware), processes the nodes in a hierarchical manner, reduces the dimensionality of the node features (channel-squeezing), and combines information from different nodes (fusion layer). This approach effectively extracts relevant information from neighboring nodes while suppressing undesired noise. Furthermore, the authors construct dynamic skeletal graphs, where the connections between nodes (representing joints in the human body) incorporate not only the fixed human skeleton topology but also the features of the nodes themselves. This allows the model to capture action-specific poses, going beyond the static structure of the human skeleton.

Building upon the potential of GCNs in the context of 3D pose estimation, Zhiming and Tang introduced a novel approach with their modulated graph convolutional network [119]. Unlike the method proposed by Zeng et al., which focuses on extracting information from neighboring nodes and building dynamic skeletal graphs, Zhiming and Tang’s model introduces weight modulation and affinity modulation. The weight modulation allows the model to learn different transformation vectors for each node, thereby learning different relations between joints. The affinity modulation adjusts the structure of the graph to incorporate additional edges beyond the skeleton structure.

Moving beyond GCN-based models, Xu et al. proposed a unique approach that combines the power of deep learning with a grammar-based model of human body configuration [120]. Their model, which takes an estimated 2D pose as input, learns a generalized 2D–3D mapping function to infer the 3D pose. The model incorporates a set of knowledge regarding human body configuration, including kinematics, symmetry, and motor coordination, enforced as high-level constraints over human poses.

In a related work, Ci et al. introduced the locally connected network (LCN) [121], which overcomes the limitations of graph convolutional networks (GCNs) by employing dedicated filters for different joints instead of shared filters. This network is jointly trained with a 2D pose estimator, allowing it to handle inaccurate 2D poses. By leveraging dedicated filters and local connectivity, the LCN enhances the accuracy of monocular 3D human pose estimation, particularly in scenarios with imperfect 2D pose inputs. By incorporating GCNs to capture the structural relationships between human body joints into their respective frameworks, DiffuPose, learning skeletal graph neural networks, and LCN showcase the effectiveness of graph-based approaches in improving the accuracy and robustness of 3D human pose estimation.

On the other hand, Gu et al. proposed a transformation method from 2D keypoints to 3D using a temporal regression network with a gated convolution module [122]. This approach focuses on incorporating temporal information to improve the accuracy of 3D pose estimation, emphasizing the importance of considering temporal dynamics in the regression process.

Another type of network to consider in the field of 3D reconstruction is the transformer network, which has gained significant popularity. GraFormer, proposed by Zhao et al. [123], exemplifies the effectiveness of this network. By combining the power of transformers and graph convolutions, GraFormer enhances the interaction among 2D keypoints and captures both apparent and hidden joint relations. The approach utilizes two core modules, GraAttention and ChebGConv block, to effectively exploit the relationships among joints and outperform previous methods. Another notable approach in the same context is MHFormer, proposed by Li et al. [124]. MHFormer employs a multi-hypothesis transformer and addresses the task by decomposing it into three stages: generating multiple initial hypothesis representations, modeling self-hypothesis communication, and learning cross-hypothesis communication to synthesize the final 3D pose. By considering multiple hypotheses, MHFormer significantly improves the robustness and accuracy of 3D human pose estimation.

#### 5.2.4. Unsupervised and Weakly Supervised Learning Methods

In contrast to the previous methods that use supervised learning, unsupervised learning methods have also been employed in the field of 3D human pose estimation. Wandt et al. introduced ElePose, which utilizes unsupervised learning with a neural network trained to recover depth [63]. This approach aims to estimate 3D human pose without relying on labeled training data. Furthermore, Yang et al. proposed CameraPose, a weakly supervised framework for 3D human pose estimation from a single image [58]. CameraPose leverages in-the-wild 2D annotations to refine the initial 2D keypoints using a refinement network module that incorporates a confidence-guided loss. This loss assigns higher weights to keypoints with higher confidence, improving the accuracy of the pose estimation. Additionally, CameraPose utilizes the camera parameters learned from the camera parameter branch to lift the refined keypoints into 3D space. This approach enables the estimation of 3D human pose from a single image. Furthermore, the paper by Luvizon et al. introduces a consensus-based optimization algorithm for multi-view predictions from uncalibrated images, offering a single monocular training procedure [125]. This algorithm allows for effective fusion of information from multiple views, improving the accuracy and robustness of 3D pose estimation.

Having discussed the various methods employed in the second stage of the two-stage pipeline for 3D pose estimation, namely, the 2D to 3D lifting, it is crucial to delve into the first stage of this pipeline: the 2D pose estimation. The accuracy and effectiveness of the 3D pose estimation heavily rely on the quality of the 2D pose estimation. Therefore, understanding the methodologies used in 2D pose estimation is essential. In the following section, we will explore the various techniques and advancements in the field of 2D pose estimation.

#### 5.2.5. Two-Dimensional Human Pose Estimation

Two-dimensional human pose estimation, the process of determining the (x, y) coordinates for each joint from a given RGB image, serves as a critical first step in the two-stage pipeline for 3D human pose estimation. The accuracy and robustness of this initial stage significantly influence the subsequent 3D pose estimation, underscoring its importance in the overall pipeline.

**Figure 3:**
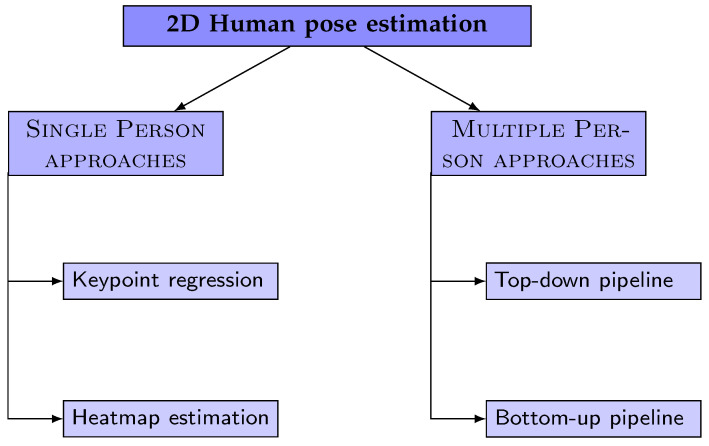
Two-dimensional human pose estimation approaches taxonomy.

The field of 2D human pose estimation has seen numerous methodologies and techniques proposed over the years, each contributing to the advancement and refinement of this crucial stage. To provide a structured and comprehensive overview of these methods, we refer to the commonly used taxonomy depicted in Figure 3, as adopted by most literature surveys. However, in our review, we will specifically focus on the most prevalent networks employed in the initial stage of estimating the 2D human pose, which precedes the reconstruction of 2D keypoints into 3D. Essentially, these methods are typically classified as ‘single-person’ approaches in survey studies, and it is this subset of methods that will form the core of our discussion.

Deep neural network (DNN) methods for predicting keypoint locations can be broadly categorized into two main groups: keypoint regression and keypoint heatmap estimation.

**Keypoint Regression Approaches:** These methods treat pose estimation as a regression problem, aiming to directly predict the Cartesian coordinates of body joints from input images. They often employ deep learning models, particularly convolutional neural networks (CNNs), to learn the complex, non-linear mappings from image features to keypoint coordinates. For instance, DeepPose, introduced by Toshev et al. [126], was one of the first methods to apply a multi-stage convolutional neural network (CNN) to this problem. The authors treated pose estimation as a regression problem and proposed to directly estimate the Cartesian coordinates of body joints using a cascade of DNN regressors. This approach was used to refine pose estimates and predict human keypoints. Carreira et al. [127] proposed an iterative error feedback-based human pose estimation system, which is repeated several times to progressively refine the pose. Zhao et al. [46] proposed a structure-aware regression approach that adopts a re-parameterized pose representation using bones instead of joints. This method effectively exploits the joint connection structure to define a compositional loss function that encodes the long-range interactions in the pose.

The main advantage of these methods is their directness in predicting keypoint locations. However, they may struggle with complex poses or occlusions due to their reliance on direct regression.

**Keypoint Heatmap Estimation Approaches:** These methods generate a heatmap for each keypoint, predicting the probability of the joint occurring at each pixel location. The pixel with the highest heatmap activation is then used as the predicted location for that joint. Tompson et al. [128] were the first to introduce keypoints heatmap estimation to address the keypoint regression problem. They proposed to jointly train a CNN and a graphical model. Lifshitz et al. [129] proposed an approach that uses information from the whole image, rather than from a sparse set of keypoint locations only. Wei et al. [130] proposed a convolutional pose machine (CPM) that consists of a sequence of CNNs that repeatedly produce 2D belief maps for the location of each part. Bulat et al. [131] proposed a method to indicate to the network where to focus, which helps guide the regression part of the network to rely on contextual information to predict the location of occluded parts. Newell et al. [132] introduced a novel CNN architecture called a stacked hourglass network. Sun et al. [133] proposed high-resolution net (HRNet), maintaining high-resolution representation through the whole process. Groos et al. [134] proposed EfficientPose, a method based on EfficientNet backbone pre-trained on ImageNet for feature extraction.

These approaches have become the most widely used techniques for coordinate representation. They are particularly effective at handling complex poses and occlusions due to their probabilistic nature, but they may be more computationally intensive than regression approaches due to the need to generate and process heatmaps.

### 5.3. Comparative Analysis of Single-Stage and Two-Stage Approaches

To conclude, 3D pose estimation is predominantly achieved through two overarching approaches: direct regression and a two-stage method that involves lifting from 2D joints. When we use these methods in real situations, we can see some clear patterns. The two-step method first estimates the pose in 2D and then uses these results to figure out the 3D pose. This way often turns out to be easier and usually provides more accurate results than the one-step direct regression approach. In fact, most leading methodologies that utilize monocular images opt for this 2D-to-3D method. The substantial advantage of this process is its ability to minimize interference from factors such as scene variation, lighting, and clothing color, that are intrinsic to RGB images.

Two-stage methods are also preferred due to their cost-effectiveness and adaptability. First, the 2D pose data, being cheaper and more abundant than their 3D counterparts, facilitates easy and efficient 2D pose prediction. Second, the methods translating 2D to 3D are less sensitive to varying scenarios and environments, thereby providing versatility. Yet, two-stage solutions are critically reliant on the performance of the 2D pose estimation. This dependency becomes a significant challenge in cases with severe occlusions in videos, where unreliable 2D keypoints can significantly impair the performance of 3D estimation. Consequently, achieving robust 3D results with noisy and occluded 2D keypoints remains a formidable task.

On the other hand, single-stage methods aim for end-to-end reconstruction, estimating 3D poses directly from RGB images without the need for an intermediate step of 2D pose estimation. These methods can leverage the rich information contained within images directly. Nevertheless, they may struggle to generalize well across varied settings such as indoor and outdoor environments, as their performance is highly dependent on the specific environment where they are deployed. Moreover, because large-scale 3D data are hard to collect and the process relies on specific tools for 3D marker tracking, the application of single-stage methods is often limited to controlled environments.

Therefore, the choice between these two approaches largely depends on the specific requirements and constraints of the project at hand. Two-stage methods might be preferred for their accuracy and adaptability, while single-stage methods could be favored for their directness and simplicity.

## 6. Three-Dimensional Multi-Person Pose Estimation from Monocular Images

The task of 3D human pose estimation becomes increasingly challenging and complex when extended to multiple persons from a monocular view. This involves detecting the poses of multiple individuals in a scene simultaneously, which introduces additional complexities beyond those of single-person pose estimation. These complexities arise from occlusions, variations in individual sizes, orientations, interactions, and the spatial relationships between individuals. Indeed, the position of one person can affect the perceived pose of another. Furthermore, the complexity increases exponentially with the number of individuals in the scene. Despite these challenges, multi-person pose estimation is of great importance in various applications, such as crowd analysis, team sports analytics, and social interaction studies.

This section aims to provide an overview of recent advances in this field, focusing on two primary types of pose estimation: relative and absolute.

### 6.1. Root-Relative Human Pose Estimation

Root-relative pose estimation, also known as person-centric coordinates, refers to a coordinate system centered around the person being detected. In this system, the positions and orientations of human joints are represented relative to the person’s root keypoint, typically the pelvis joint. This coordinate system is commonly used in monocular view scenarios. We organize the approaches based on the pipeline they follow: top-down approaches, bottom-up approaches, and unified single-stage approaches.

**Top-down approaches** involve two steps; initially detecting each person in the image, followed by estimating the 3D pose for each detected person. For example, Zanfir et al. [135] proposed a system to estimate the 3D pose and body shape of multiple people. Their system combined a single-person pose estimation model based on the deep multitask human sensing network and semantic feedback, with additional constraints such as ground plane estimation, mutual volume exclusion, and joint inference for all people in the scene. The pose and shape parameters of the SMPL human body model were then refined using non-linear optimization based on semantic fitting. Another example of a top-down approach is the pose estimation and dectection anchor-based network (PandaNet) [136] proposed by Benzine et al. PandaNet is a single-shot anchor-based approach that performs bounding box detection and 2D and 3D pose regression in a single forward pass, without requiring any post-processing to regroup joints. To handle overlapping people, PandaNet uses a pose-aware anchor selection strategy and optimizes weights associated with different people scales and joint coordinates to address the inherent imbalance among varying people sizes in images.

**Bottom-up approaches** aim to estimate all individuals’ poses and then associate each pose to a specific person. A good example is the method proposed by Mehta et al. [137]. This approach applies temporal and kinematic constraints in three steps to predict occlusion-robust pose maps (ORPMs) and part affinity fields, a technique that was previously introduced by Cao et al. [138]. This method produces multi-person 2D joint locations and 3D pose maps in a single shot.

Finally, **unified single-stage methods** aim to solve the task by directly regressing the 3D poses in a single step or stage, rather than breaking it down into several sub-tasks. An example of such a method is the localization–classification–regression (LCR) network, commonly referred to as LCR-Net [139], and its subsequent version, LCR-Net++ [140]. The LCR-Net architecture is an end-to-end system comprising three main stages that share convolutional feature layers and are jointly trained. The first stage, localization, generates a set of pose proposals in the image using a fixed set of anchor poses. These poses are then transformed into bounding boxes with the aid of a region proposal network (RPN). Following localization, the classification stage takes over. It scores the different pose proposals and predicts the closest anchor-pose for each bounding box. The set of K-poses used in this process are obtained from a motion capture (MOCAP) dataset, ensuring a wide variety of potential poses. The final stage involves a regressor, which refines the pose proposals in both 2D and 3D dimensions. This refinement process is crucial for achieving accurate pose estimations. The final pose estimation is then obtained by integrating neighboring pose hypotheses, providing a comprehensive and precise pose estimation.

Furthermore, Sun et al. [91] introduced a model named BEV (bird’s-eye view) for this task. This model directly regresses the pose and shape of all the people in the scene, as well as their relative depth. The end-to-end design of BEV simplifies the process and enhances efficiency, making it a single-shot method that is end-to-end differentiable. The model introduces a novel bird’s-eye view representation, enabling powerful 3D reasoning that reduces the monocular depth ambiguity. By exploiting the correlation between body height and depth, BEV learns depth reasoning from complex in-the-wild scenes, leveraging relative depth relations and age group classification.

Although relative pose estimation is common in monocular view scenarios, understanding multi-person behavior and scene visualization often requires considering the distance between individuals. This is where absolute pose estimation becomes critical. However, only a few works have tackled the problem of camera-centric multi-person 3D pose estimation from a monocular RGB image or video.

### 6.2. Absolute Human Pose Estimation

On the other hand, absolute pose estimation, known also as camera-centric coordinates, refers to a coordinate system centered around the camera. Here, the positions and orientations of human joints are represented relative to the camera’s viewpoint, making it most suitable for real-world applications.

**Figure 4:**
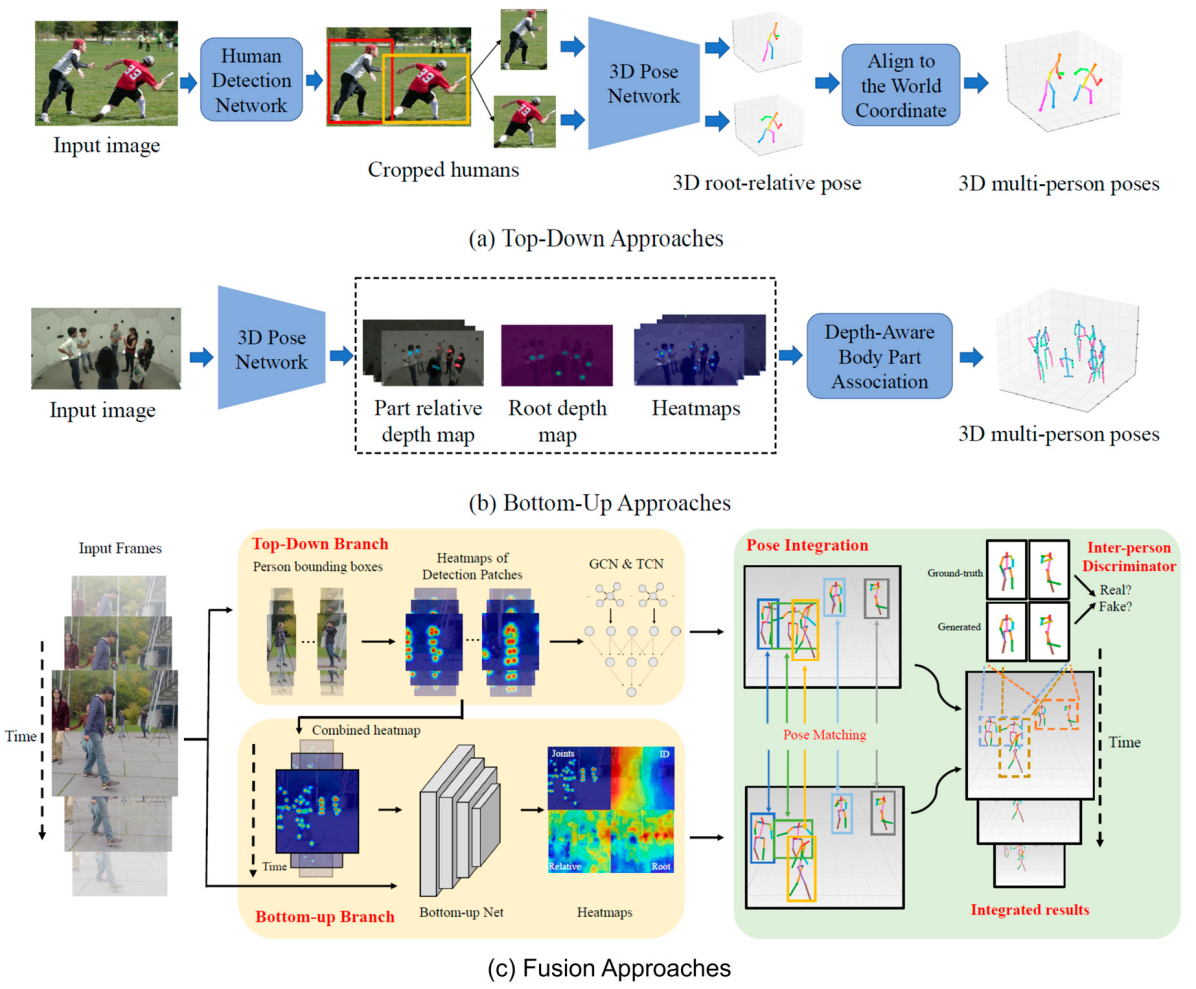
Absolute human pose estimation approaches.

In terms of categorizing approaches, we propose to classify them into four distinct categories. These classifications represent the primary taxonomy used in relative pose estimation approaches. However, we identify an additional category specific to absolute pose estimation, known as fusion approaches. These fusion methods seamlessly integrate aspects from both top-down and bottom-up approaches, thereby providing more nuanced and robust solutions for multi-person 3D pose estimation. Figure 4 illustrates the network architecture of each category.

#### 6.2.1. Top-Down Approaches

Top-down approaches to 3D multi-person pose estimation typically involve a pipeline consisting of human detection, absolute 3D human root localization, and root-relative 3D single-person pose estimation modules. For example, Moon et al. [141] employed this pipeline using the RootNet model for absolute 3D human root localization and the PoseNet model [142] for root-relative 3D single-person pose estimation. AnimePose [4] generated a 3D animation of several people from a 2D video in a top-down manner using a depth map to locate 3D poses, a 3D IOU (intersection over union) metric at the top of the 2D pose prediction for tracking. A multi-person trajectory estimate was used to continue the tracking process in closed frames. The end-to-end HDNet (human depth estimation network) [143] follows the same pipeline. It estimates the depth of a person in an image by using a combination of a feature pyramid network [144] for general feature extraction and separated multi-scale feature extraction for pose and depth estimation, and a graph neural network to propagate and aggregate features for the target person’s depth estimation. The estimated depth is represented as a bin index and can be transformed into a continuous value using a soft-argmax operation. Similar to the above methods for depth estimation, HMORs (hierarchical multi-person ordinal relations) [145] employs an integrated top-down model to estimate human bounding boxes, depths, and root-relative 3D poses simultaneously, with a coarse-to-fine architecture that, instead of using image features as the above methods for depth estimation, hierarchically estimates multi-person ordinal relations of depths and angles which captures body-part and joint-level semantics while maintaining global consistency to improve the accuracy of depth estimation. The framework proposed for 3D multi-person pose estimation in [146] combines GCNs and TCNs to estimate camera-centric poses without requiring camera parameters. It includes GCNs that estimate frame-wise 3D poses and TCNs that enforce temporal and human dynamics constraints to estimate person-centric with a joint-TCN and camera-centric 3D poses across frames with a root-TCN. Reddy et al. [147] focus on 3D pose estimation and tracking of multiple people in video frames taken from single or multiple cameras. They propose a top-down approach, TesseTrack, that simultaneously reasons about multiple individuals’ 3D body joint reconstructions and associations in space and time in a single end-to-end learnable framework. TesseTrack operates in a voxelized feature space and consists of several steps: person detection, short-term person-specific representation computation, time linking, and 3D pose deconvolution. This method treats the 2D pose estimation, 2D-to-3D lifting, and 3D pose tracking in a joint spatio-temporal framework, contrasting traditional piece-wise strategies. In a weakly supervised paradigm, Cong et al. [57] present a top-down approach for 3D multi-person pose estimation in large-scale scenes. The method uses a monocular camera and LiDAR sensor data, exploiting the benefits of multi-modal input data and temporal information. It first detects individuals in the scene and then estimates their 3D pose using fused data from the image and point cloud. This method is designed to be easy to deploy and insensitive to light conditions. Temporal information is used to guide the network in learning natural and coherent human motions without requiring precise 3D annotations.

While these methods have shown promising performance, they can be affected by inter-person occlusions and close interactions because they process each person independently. Furthermore, they ignore the global context since they process the cropped image.

#### 6.2.2. Bottom-Up Approaches

Bottom-up approaches to 3D multi-person pose estimation first generate all body joint locations and depth maps. They then associate body parts to each person based on the root depth and the relative depth of the parts. For instance, Fabbri et al. [148] proposed a method that estimates volumetric heatmaps in an encoder–decoder manner. They first produce compressed volumetric heatmaps, which are used as ground truth, and then decompress them at test time to re-obtain the original representation. Zhen et al. [149] estimated 2.5D representations of body parts and reconstructed 3D human pose in a single-shot bottom-up framework. Zhang et al. [150] proposed a unified bottom-up model which integrates 2.5D pose representation and depth estimation. The model utilizes a structured 2.5D pose estimation for recognizing inter-person occlusion, and an end-to-end geometry-aware depth reasoning method. It infers camera-centric root depths using 2.5D pose and geometry information and then refines the 2.5D pose estimation learning using root depths. The method proposed by Benzine et al. [151] extends the stacked hourglass network to handle multi-person scenarios without requiring bounding boxes. Using an associative embedding method, it performs joint grouping and human pose estimation. Occlusions and cropping are handled by storing joint coordinates redundantly in multiple 2D locations in the occlusion robust pose maps (ORPMs). XNect [152], on the other hand, uses a sequential multi-stage approach. Stage I employs a convolutional neural network to process the entire input frame, generating 2D body joint heatmaps and an intermediate 3D pose encoding per detected joint. After grouping 2D joint detections into individuals, stage II uses a fully connected neural network to decode the input into a full 3D pose per individual. Stage III subsequently performs sequential model fitting on the live stream of predictions, achieving temporally coherent motion capture results. Even though XNect primarily adopts a bottom-up approach, the second stage introduces a top-down component by reasoning over all joints to produce a full pose.

These bottom-up approaches offer alternatives to top-down methods, potentially better handling inter-person occlusions and close interactions. It should be noted that these classifications are often non-binary, and many papers utilize elements from multiple categories.

#### 6.2.3. Fusion Approaches

Fusion, or hybrid, approaches integrate elements from both top-down and bottom-up methods, thus leveraging the strengths of each to improve overall performance. Typically, such methods might initially detect individuals, estimate their joint positions, and finally refine the assignment of joints to individuals based on the initial detections.

Several works have recently succeeded in integrating the top-down and bottom-up pipelines to navigate inter-person occlusion and close interaction challenges. For example, the TDBU_Net framework [51] combines a top-down network that estimates joints for all persons in an image patch with a bottom-up network that uses human-detection-based normalized heatmaps to adeptly manage scale variations. The 3D pose estimates generated by these two distinct networks are integrated via a third network to produce the final 3D poses.

In another hybrid approach introduced in a subsequent study [52], a fusion network is employed to blend top-down and bottom-up networks, enhancing the robustness of pose estimation from monocular videos. This fusion network unifies the 3D pose estimates to generate the final 3D poses. This method stands out due to its integrated approach and its utilization of test-time optimization to address disparities between training and testing data. It incorporates a two-person pose discriminator and employs a semi-supervised strategy to mitigate the scarcity of 3D ground truth data.

#### 6.2.4. Unified Single-Stage Approaches

These methods perform all the steps in the process, including 2D detection, 3D conversion, and temporal information fusion, in a single forward pass without the need for separate explicit stages.

In their paper, Jin et al. [153] introduce the decoupled regression model (DRM), a unified model that simultaneously conducts 2D pose regression and depth regression. This model’s key contributions include a novel decoupled representation for 3D pose, a 2D pose-guided depth query module (PDQM), and a decoupled absolute pose loss (DAPL) strategy. These innovations enable DRM to perceive scale information of instances adaptively and improve the accuracy of depth prediction.

In parallel, Wang et al. [92] present a distribution-aware single-stage (DAS) model. This approach bypasses traditional multi-stage methods and uses a unified approach to represent 3D poses. By incorporating a 2.5D human center and 3D center-relative joint offsets, DAS simplifies the 3D pose estimation problem into a single-pass solution. DAS demonstrates potential for higher efficiency and comparable accuracy compared to two-stage methods.

Zhang et al. [154] propose another significant approach within this category, the ray-based 3D (Ray3D) method. This method uses a single unified model to carry out all necessary steps for 3D human pose estimation. It directly transforms 2D keypoints into 3D rays, taking both camera intrinsic and extrinsic parameters into account. Furthermore, it fuses temporal data, all within a single comprehensive model.

Although these approaches are not typically classified as ‘top-down’ or ‘bottom-up’, they can be regarded as a variant of a top-down approach. This is due to their simultaneous prediction of 2D poses and depth information from the input image, thereby eliminating the need for a separate stage to associate detected joints into individual poses.

### 6.3. Analytical Comparison of Multi-Person Pose Estimation Methods

In this section, we provide an overview of the complex field of 3D multi-person pose estimation from monocular images. This task brings substantial challenges to the forefront, particularly the issues of occlusion and interaction between individuals.

We emphasized the distinction between relative and absolute poses in our discussion. Relative poses allow us to examine the spatial relationships between various body joints in 3D space, providing crucial information about the posture and movement of individuals. In contrast, absolute poses offer an accurate depiction of these joints’ exact locations in the world coordinate system. For real-world applications, understanding the exact placement of individuals within a scene is paramount, and thus, absolute poses hold more significance. As such, we have focused more on the approaches dealing with absolute pose estimation in our review. Furthermore, relative pose estimation can sometimes resemble applying single-person 3D pose estimation multiple times for each person present in a scene. However, this approach does not necessarily consider the interactions between different individuals or their precise locations within the global environment. Hence, while it may provide useful posture information, it lacks the context provided by absolute pose estimation methods.

We also inspected and compared different methodologies for pose estimation, primarily top-down, bottom-up, and unified single-stage approaches. Top-down techniques sequentially detect individuals in a scene before estimating their respective poses, while bottom-up methods detect all body joints within the scene before associating them to form individual poses. Although both these methods hold their own strengths, they generally necessitate separate stages for detection and pose estimation, leading to efficiency issues. The quantitative comparisons of these methodologies, particularly in terms of root-relative and absolute 3D multi-person pose estimation, are detailed in Table 3. Emerging unified single-stage methods such as DAS, DRM, and Ray3D present potential solutions to these challenges. They propose to estimate 2D poses and depth information simultaneously from the input image in a single-pass solution, achieving a promising balance between accuracy and computational efficiency.

Nonetheless, it is important to highlight the significant performance gap between single-camera and multi-camera 3D pose estimation methods. The extra views you get from using multiple cameras give them a clear advantage in creating accurate 3D poses, particularly in camera-centered coordinates. Therefore, the big challenge is to improve methods that only use one camera so they can perform as well, or even better, than methods using multiple cameras.

## 7. Three-Dimensional Human Pose Estimation from Monocular Videos

The task of understanding human activity, including pose estimation, often involves processing a sequence of images or a video. A variety of approaches have been developed to leverage temporal information, irrespective of the specific methodology used for 3D pose estimation.

The approaches applied on video sequences generally follow the same methodologies used in single-frame pose estimation. Some methods directly regress the 3D poses in a one-stage pipeline [71,89], while others follow a two-stage pipeline, first locating the joints in 2D and then lifting them to 3D [140,155,156,157].

However, when working with sequences of images, maintaining temporal consistency is key. In this section, we review the approaches that incorporate temporal information from video sequences. Video-based methods face the challenge of maintaining the temporal consistency of the estimated poses, which can be influenced by rapid movements, occlusions, and changes in the scene. Additionally, tracking is a crucial phase when dealing with videos.

Despite these challenges, significant progress has been made in recent years, largely due to the advancement of deep learning techniques. Recurrent neural networks (RNNs), particularly long short-term memory (LSTM) networks, have been employed to model the temporal dependencies in video sequences, resulting in more consistent and smooth pose estimations. More recently, graph convolutional networks (GCNs) and transformer networks have been introduced to model the spatial relationships among the joints and the temporal relationships among the frames, respectively.

### 7.1. Methods Based on LSTM

Long short-term memory networks (LSTMs) are a specialized type of recurrent neural network (RNN) designed to retain information over extended periods and learn long-term dependencies [158]. This makes them particularly effective for tasks requiring sequential data, such as time-series prediction, natural language processing, and, in the context of this survey, 3D human pose estimation from video.

In the realm of 3D human pose estimation, several researchers have leveraged the capabilities of LSTM networks. For example, Lee et al. [157] proposed an LSTM-based deep learning architecture called propagating LSTM networks (p-LSTMs). In this architecture, each LSTM is connected sequentially to reconstruct 3D depth from the centroid to edge joints through learning the intrinsic joint interdependency (JI). The first LSTM creates and reconstructs the seed joints of 3D pose into whole-body joints through the connected LSTMs. The p-LSTMs were connected in series to elaborate the 3D pose while transferring the depth information, referred to as pose depth cues. In another work, AnimePose [4] utilized Scene-LSTM to estimate the temporal trajectory of the person and track overlapping poses in occluded frames. The authors predicted the missing poses in previous frames, plotted the trajectory of each keypoint, and then estimated the position of joints in subsequent frames. Zhang et al. [159] proposed a spatial-temporal convolutional long short-term memory model (ST-CLSTM) with two parts to capture spatial features and temporal consistency among frames. The model’s weights were updated using temporal consistency and spatial losses between the estimated and the ground truth depths, computed by a 3D convolutional neural network (3D CNN). To maintain temporal consistency among the estimated depth frames, the authors used generative adversarial networks (GANs) as a temporal consistency loss. In this setup, a 3D CNN acted as a discriminator to output the temporal loss from the estimated and ground truth depth sequences, while the ST-CLSTM served as the generator. Lastly, Liu et al. [48] proposed an end-to-end trainable recurrent neural network for full pose mesh recovery from videos. Using a long short-term memory (LSTM) structure, the model explicitly learns to model temporal coherence and imposes geometric consistency over the recovered meshes. The authors also introduce an attention mechanism in the recurrent framework, which achieves both precise shape estimation and coherent temporal dynamics in the resulting 3D human pose sequences.

### 7.2. Methods Based on TCNs

Temporal convolutional networks (TCNs) are a type of neural network designed for sequence modeling. They use dilated convolutions and causal connections to capture long-range dependencies in time-series data. TCNs have been shown to outperform recurrent architectures like LSTMs on a variety of tasks, due to their ability to handle longer sequences and their greater computational efficiency. In the context of 3D human pose estimation from video, TCNs can be used to model the temporal dependencies between different frames, which can help to improve the accuracy of the pose estimation.

In the PoseNet3D study [61], the authors utilized dilated convolutions to model temporal dynamics. This method facilitates feedback at each time-step and effectively bypasses common issues associated with LSTM/RNN usage. The strategy of employing residual connections and dilated temporal convolutions over 2D keypoints is also seen in other research [156,160], specifically, the TCN [161] is used to lift 2D keypoints to 3D joints. The TCN architecture is a one-dimensional fully convolutional network that integrates causal dilated convolutions. Each hidden layer in the TCN has the same length as the input layer, enabling the architecture to map an input sequence of any length to an output sequence of the same length, akin to an RNN. In their work, Pavllo et al. [156] start with predicted 2D keypoints for unlabeled video, estimate 3D poses, and then use a semi-supervised training method to back-project 3D data to the input 2D keypoints. This approach is unique in its use of a semi-supervised training method and back-projection of 3D data. Chen et al. [162] extended the work of [156] by adding a bone-direction module and a bone-length module to ensure human anatomy temporal consistency across video frames. The authors propose a joint shift loss to ensure consistency between predicted bone lengths and directions. Liu et al. [155] proposed a graph attention spatio-temporal convolutional network (GAST-Net), which includes a dilated temporal model to handle long-term patterns in multi-frame estimation. GAST-Net also incorporates graph attention blocks, including a local spatial attention network to model the hierarchical and symmetrical structure of the human skeleton, and a global spatial attention network to extract global semantic information for better encoding of the human body’s spatial characteristics. This approach is distinct in its use of graph attention blocks and a global spatial attention network. Lastly, in Ref. [163], the authors proposed an occlusion-guided framework that estimates more accurate poses from 2D skeletons with missing joints as input. Missing joints are handled by introducing guidance that provides extra information about the absence or presence of a joint. Temporal information is exploited to better estimate the joints, using a temporal dilated convolutional neural network (CNN). This approach is unique in its focus on handling missing joints and using guidance to provide extra information about the absence or presence of a joint.

While TCNs have proven effective in modeling temporal dependencies in video sequences, another important aspect of 3D human pose estimation is the spatial relationships among the joints. This is where GCNs come into play.

### 7.3. Methods Based on GCNs

Graph convolutional networks are a type of neural network designed to work with graph-structured data, making them particularly suitable for tasks that involve spatial relationships. In the previous Section 7.2, we introduced GAST-Net, which combines TCNs and GCNs. Although we discussed it there, we can also include it in this subsection. The graph attention blocks employed in GAST-Net capture the symmetrical hierarchy of 2D skeleton and global postural constraints. These blocks are effectively combined with temporal dependencies to address depth ambiguity and overcome self-occlusion. Likewise, Cheng et al. [146] also presents a method that combines GCNs and TCNs but for 3D multi-person pose estimation in monocular videos. The framework introduces two types of GCNs: a human-joint GCN and a human-bone GCN. The human-joint GCN is based on a directed graph that uses the 2D pose estimator’s confidence scores to improve pose estimation results. The human-bone GCN models the connections between bones, providing additional information beyond human joints. These two GCNs work together to estimate spatial frame-wise 3D poses and can use both visible joint and bone information in the target frame to estimate occluded or missing human-part information. Wang et al. [164] proposed the simplified-attention enhanced graph convolutional network (SaEGC-Net), which effectively models joint dependencies through two distinct hierarchies of human structure. Attention mechanisms are integrated into GCNs to establish stronger motion constraints. The SaEGC-Net is a U-shaped network consisting of three stages: downsampling, upsampling, and merging. The network accepts consecutive 2D sequences as input and produces 3D poses as output. The network architecture includes features of varying sizes, with the horizontal size representing the number of channels, the vertical size representing the temporal length, and the depth of the cuboid representing the number of joints. A one-hop spatio-temporal graph convolution (ST-GC) module, followed by a two-hop ST-GC module, forms a single cascaded spatio-temporal graph convolution (CST-GC) block. The ST-GC modules in the upsampling stage utilize one-hop graphs. In a related study, Zhang et al. [165] introduced an approach that incorporates uncertainty awareness through the use of evidential deep learning (EDL). This approach integrates uncertainty modeling into depth prediction and employs a probabilistic representation to account for the distribution uncertainty of 2D detection. To further enhance the performance of their model, the authors developed an encoder that merges a graph convolutional network (GCN) and a transformer. The GCN is utilized to model the spatial relationships among the input 2D keypoints, while the transformer is used to model the temporal relationships among the input frames. This fusion of methodologies enables the encoder to capture both spatial and temporal correlations among the input keypoints, leading to more accurate pose estimation.

This work highlights the versatility and potential of transformer networks, which were originally introduced for natural language processing tasks. Due to their ability to model long-range dependencies, transformers have shown great promise in various domains, including pose estimation. In the following section, we will delve into papers that have utilized and adapted transformer networks for the task of 3D human pose estimation.

### 7.4. Methods Based on Transformers

Transformer-based methods constitute a category of deep learning architectures that utilize self-attention mechanisms to handle sequential data, including text or images. Transformers can be directly employed on image patches or can be integrated with convolutional layers to discern long-range dependencies and spatial relationships among features. The primary advantage of Transformer networks is their capacity to model global dependencies among all elements in a sequence, irrespective of their relative positions. This is a contrast to TCNs, which are primarily designed to handle temporal dependencies within a certain range. To reconstruct 3D pose from a long sequence of predicted joint locations, Li et al. [166] introduced a strided transformer, an improved transformer-based architecture. The authors employ a vanilla transformer encoder (VTE) to model long-range dependencies of 2D pose sequences and a strided transformer encoder (STE) to reduce the redundancy of the sequence and aggregate information from local contexts. The proposed method also includes a full-to-single supervision scheme to impose extra temporal smoothness constraints during training. Building on this, the MixSTE approach proposed by Zhang et al. [167] employs a temporal transformer block and a spatial transformer block alternately to achieve better spatio-temporal feature encoding. This dual-block structure allows the network to effectively capture both the individual movements of each joint and the relationships between different joints, demonstrating the versatility and effectiveness of transformer-based models in this domain. Similarly, the PoseFormer model introduced by Zheng et al. [168] is a purely transformer-based approach that designs a spatial-temporal transformer structure to model the human joint relations within each frame as well as the temporal correlations across frames. This approach further underscores the potential of transformer networks in capturing both spatial and temporal dependencies for 3D human pose estimation. Moreover, the work of Wei et al. [47] introduced a motion pose and shape network (MPS-Net) that leverages visual cues observed from human motion to adaptively recalibrate the range that needs attention in the sequence to better capture the motion continuity dependencies. Meanwhile, Li et al. [124] proposed a multi-hypothesis transformer (MHFormer) designed to learn spatio-temporal representations of multiple plausible pose hypotheses and gradually communicate across them to synthesize a more accurate prediction. This model showcases the potential of transformer networks in handling multiple hypotheses and synthesizing them for more accurate 3D pose estimation. On the other hand, the P-STMO (pre-trained spatial temporal many-to-one) model introduced by Shan et al. [70] presents a unique two-stage approach to 3D human pose estimation from video, leveraging pre-training techniques and a simplified model structure to effectively capture both spatial and temporal dependencies in human movement. The process is divided into pre-training and fine-tuning stages, each designed to capture different aspects of spatial and temporal information. In the pre-training stage, a self-supervised sub-task called masked pose modeling is proposed, where human joints in the input sequence are randomly masked in both spatial and temporal domains. A general form of denoising autoencoder is used to recover the original 2D poses, enabling the encoder to capture spatial and temporal dependencies. In the fine-tuning stage, the pre-trained encoder is loaded into the STMO model and fine-tuned. The encoder is followed by a many-to-one frame aggregator designed to predict the 3D pose in the current frame. This two-stage strategy allows the encoder to capture 2D spatial temporal dependencies in the pre-training stage and extract 3D spatial and temporal features in the fine-tuning stage. The P-STMO model also introduces a temporal down-sampling strategy on the input side to reduce data redundancy. In a different paradigm, Gong et al. [69] proposed a self-supervised approach that generates 2D–3D pose pairs for augmenting supervision through a self-enhancing dual-loop learning framework. This approach underscores the potential of transformer networks in self-supervised learning scenarios for 3D human pose estimation, demonstrating the versatility and adaptability of transformer-based methods in this domain.

### 7.5. Unified Frameworks for Real-Time Applications

Unified frameworks are designed to handle all aspects of a particular task within a single, integrated system. These frameworks are typically optimized for efficiency and speed, making them suitable for real-time applications where quick processing and response times are critical.

In the context of 3D human pose estimation, a unified framework encompasses various essential components within a single, integrated system. This comprehensive approach combines the functionalities of detecting and tracking human figures in video, estimating 2D joint positions, lifting these 2D positions to 3D, and smoothing or refining the pose estimates over time. Additionally, the framework may also incorporate the capability of absolute depth estimation, further enhancing the accuracy and completeness of the pose estimation process. By integrating all these components into a unified structure, the framework can efficiently and rapidly process data, making it well-suited for real-time applications where quick and accurate results are crucial.

Luvizon et al. [89] proposes a multitask network architecture that jointly estimates 2D/3D human poses and recognizes associated actions through regression. This approach follows a single-stage pipeline and benefits from using images “in the wild” with both 2D annotated poses and 3D data. This has been proven to be a very efficient way to learn visual features. Similarly, the work [88] presents SSP-Net, an end-to-end trainable architecture for regressing the pose in multiple scales in a sequential coarse-to-fine manner. SSP-Net consists of an entry flow that extracts preliminary feature maps from input images, and a sequence of CNNs comprising prediction blocks connected by alternating downscaling and upscaling units. Each prediction block outputs a supervised pose prediction that is further refined by subsequent blocks and units.

In contrast, Ref. [169] introduces a two-stage pipeline and presents a unified framework that combines YOLOv5, HRNet, and TCN for real-time 2D/3D human pose estimation. In the work presented in [170], a similar pipeline was pursued, but it was expanded by incorporating a root depth estimator, a feature not present in the approach outlined in [169]. This incorporation enables the system to derive camera-centric coordinates and integrates YOLOv3 for human detection, HRNet as the 2D pose estimator, and GAST-Net for 3D root-relative pose reconstruction. The resulting system, named Root-GAST-Net, is specifically tailored to tackle the challenges associated with recovering the 3D absolute poses of multiple individuals from a monocular perspective.

Adopting a top-down approach similar to Refs. [169,170], Dong et al. [171] proposed an approach for multi-person 3D pose estimation. However, a notable distinction lies in their consideration of multiple views instead of a monocular view. They employed a multi-way matching algorithm to cluster detected 2D poses across all views, encoding the 2D poses of the same individuals from different views into resulting clusters and establishing consistent correspondences across keypoints. This key difference allows the approach presented by Dong et al. [171] to take advantage of the multi-view setup, leading to potentially improved accuracy and robustness in multi-person 3D pose estimation. Similarly, in the context of multi-view setups, the work proposed in [172] presents an approach for performing 3D pose estimation of multiple individuals from a few calibrated camera views. Their architecture aggregates feature maps from a 2D estimator backbone, creating a comprehensive representation of the scene. Subsequently, this representation is enhanced by a fully convolutional volumetric network and a decoding stage, which effectively extracts skeletons with sub-voxel accuracy.

The advantage of unified frameworks lies in their ability to achieve superior performance compared to a collection of separate models. This is because each component within the framework can be optimized to work seamlessly with the others, resulting in enhanced overall performance. Additionally, unified frameworks offer greater efficiency by eliminating the need for data to be passed between separate models or systems, making them well-suited for real-time applications that require quick processing. As an example, in [170], the effectiveness of the unified framework was demonstrated, attaining an impressive 15 frames per second (fps) on the Nvidia GeForce GTX 1080. Emphasizing the utilization of high-performance materials and FP16 precision can lead to even greater enhancements in processing speed.

### 7.6. Performance and Complexity Analysis of Video-Based Approaches

When a video sequence is available, researchers often employ temporal models to map and model the connections between keypoints across images, effectively reducing the search space. As we have discussed, various networks such as LSTM and TCN are used to preserve information over extended periods.

In terms of performance, LSTM-based methods have demonstrated robustness in managing temporal dependencies in video sequences, making them suitable for pose estimation tasks involving sequential data. However, they may encounter difficulties with long-term dependencies due to the vanishing gradient problem. Conversely, TCN-based methods have exhibited superior performance in capturing long-range dependencies, thanks to their dilated convolutions and residual connections.

GCN-based methods excel in scenarios where pose estimation involves a graph structure, such as a human skeleton. These GCN networks are utilized for spatial relationships. They have demonstrated high accuracy in such scenarios due to their ability to capture the dependencies between different joints. Additionally, transformers, with their attention mechanism, are employed to discern long-range dependencies and spatial relationships among features. They have shown promising results in pose estimation tasks by effectively managing long-range dependencies and occlusions.

In terms of complexity, LSTM and TCN-based methods have relatively lower computational requirements compared to GCN and transformers. The latter methods, while powerful, often require significant computational resources due to their complex architectures. LSTM and TCN-based methods are also easier to implement and train, making them more accessible for researchers and practitioners.

Unified frameworks, designed to integrate the advantages of various methods, have demonstrated high accuracy and robustness across different scenarios. However, their performance is heavily contingent on the specific combination of methods employed within the framework. These frameworks are typically more complex to implement and demand substantial computational resources. Additionally, they necessitate meticulous design and tuning to ensure the harmonious operation of the integrated methods. In the case of multi-person scenarios, tracking must be applied to ensure temporal consistency of poses.

In conclusion, Table 4 offers a general comparison of the methods used for 3D human pose estimation in video sequences. The choice of network should be guided by careful consideration of the task’s specific requirements, the computational resources available, and the balance between complexity and performance. Additionally, Table 5 details the mean per-joint position error (MPJPE)—a key metric for accuracy—in notable examples from each method category, tested on the Human3.6M dataset with a sequence of ground truth 2D poses. These data offer a clearer visualization of each method’s performance.

## 8. Three-Dimensional Human Pose Estimation from Multi-View Cameras

In the field of 3D human pose estimation, the use of multiple camera views has emerged as a powerful strategy to overcome the inherent challenges associated with the loss of depth information during the projection from the 3D world to the 2D image plane. Multi-view approaches offer a more comprehensive understanding of spatial structure, enabling more accurate pose estimation by capturing different perspectives of the subject.

Multi-view approaches in 3D human pose estimation can be considered as weakly supervised human pose estimation. They aim to obtain ground truth annotations for monocular 3D human pose estimation. One approach is the use of weak supervision transfer learning, as explored by Zhou et al. [175], where training data with mixed 2D and 3D labels are employed. The authors introduced a 3D geometric constraint to regularize the predicted 3D poses on images with only 2D annotations. Rhodin et al. [176] proposed replacing most of the annotations with multiple views to train the system to predict the same pose across all views. Zhou et al. [177] exploited different views to predict 3D keypoints of an object on unlabeled instances.

However, these methods often require synchronized and calibrated cameras, a requirement that may not always be feasible, particularly in uncontrolled environments. Multi-view camera approaches for 3D human pose estimation also typically follow either a single-stage [90,178] or a two-stage pipeline [56,125,179]. These multi-view approaches are particularly crucial when the goal is to estimate poses in camera coordinates, as they can leverage the additional spatial information provided by multiple views to generate more accurate and robust estimations in the camera’s coordinate system. This aspect becomes especially important in multi-person scenarios.

As an example, the work by Luvizon et al. [125] proposes a method for 3D human pose estimation in camera coordinates. This method effectively combines 2D annotated data and 3D poses, offering a straightforward multi-view generalization. The paper also presents a consensus-based optimization algorithm for multi-view predictions from uncalibrated images, predicting each body joint in image-pixel coordinates and in absolute depth, orthogonal to the image plane.

Transformer-based models have gained popularity in this field due to their ability to handle complex spatial relationships and their capacity for parallel computation, which is beneficial for processing multi-view data. Zhang et al. [90] propose a transformer-based model, the multi-view pose transformer (MvP), which directly regresses the 3D poses by representing query embedding of multi-person joints. The multi-view information for each joint is fused by a geometrically guided attention mechanism, called projective attention. This mechanism effectively harnesses the additional depth provided by multi-view data, providing complementary information that aids in the estimation of absolute multi-person poses.

Similarly, the TransFusion framework introduced by Ma et al. [180] employs a transformer model and introduces the concept of an epipolar field to encode 3D positional information, allowing for efficient encoding of correspondences between pixels of different views.

The use of multi-view approaches is not limited to images. Dong et al. [171] proposes a fast and robust approach to solve the problem of multi-person 3D pose estimation and tracking from multiple view videos. The approach uses a multi-way matching algorithm to cluster the detected 2D poses in all views. Each resulting cluster encodes 2D poses of the same person across different views and consistent correspondences across the keypoints, from which the 3D pose of each person can be effectively inferred.

Real-time applications have also been explored. Zhang et al. [179] introduce an end-to-end point-to-pose mesh fitting network (P2P-MeshNet) to directly estimate the body joint rotations from multi-view OpenPose 2D joint locations in real time, with a performance of 100 frames per second.

In the realm of self-supervised learning, Wandt et al. [56] propose CanonPose, a framework that uses unlabeled multi-view data to infer 3D poses. The framework of CanonPose involves multiple neural networks where each sub-network produces a 3D pose in a canonical rotation. The outputs across different views are combined to produce the final pose. This method does not require prior knowledge about the scene, 3D skeleton, or camera calibration, and integrates the confidences from the 2D joint estimator into the training pipeline. The outputs across different views are combined to produce the final pose.

In the same learning paradigm but applied to videos, Gholami et al. [181] propose a learning-based method to overcome the limitations of classical triangulation. This approach trains on uncalibrated multi-view videos without needing 3D annotations or calibrated cameras, and applies to single videos at inference time.

### Challenges of Multi-View Camera Systems

To conclude, multi-view camera systems have greatly advanced 3D human pose estimation, introducing new methods for improving accuracy and expanding potential applications. However, these techniques face challenges.

They necessitate specific camera configurations, which may not be accessible or practical in numerous real-world contexts. Furthermore, the processing time required for information derived from these systems can be substantial. This lack of speed can render them inefficient for scenarios requiring immediate results, such as real-time tracking or instantaneous feedback. Spatial constraints also pose an issue. Cameras need to be stationed at varying angles around the subject, a requirement that might not be achievable in compact or cluttered spaces, thereby restricting the areas where these systems can be used. Cost is another potential obstacle. Implementing multi-view systems can be expensive due to the need for multiple cameras and their particular setup, potentially limiting their usage to those who can bear such costs.

While multi-view camera systems have undeniably pushed 3D human pose estimation to new heights, these constraints reduce their practicality for various real-world scenarios.

## 9. Common Databases and Evaluation Metrics for 3D Human Pose Estimation

Table 6 provides a list of commonly used datasets for 3D human pose estimation, while Table 7 details the specific evaluation metrics employed in these studies.

## 10. Conclusions

In conclusion, our survey has provided a comprehensive overview of various approaches and techniques developed for 3D human pose estimation from monocular images, videos, and multi-view images. We have thoroughly categorized and analyzed these methods, taking into account factors such as the number of people involved, the input data, and approaches for interpreting body structure.

The advent of deep learning has led to significant advancements in the field of 3D human pose estimation, enabling more accurate and precise results.

However, it is important to note that despite these advancements, there are still limitations in the real-life application of these methods. Challenges persist in accurately estimating poses for multiple individuals or in challenging conditions, such as outdoor environments, scenarios involving rapid movements, or situations where the subject is either too small or far from the camera. These challenges are particularly evident in monocular camera setups, which can pose additional difficulties in obtaining absolute results.

To further improve the practicality and effectiveness of 3D human pose estimation techniques, future research should focus on addressing these challenges and developing robust algorithms that can handle complex real-world scenarios.

## Figures and Tables

**Table 1:** Learning methods.

Learning Paradigm	Description	References
Supervised Learning	Refers to the use of labeled training data where each input sample (e.g., an image or a video frame) is paired with its corresponding ground truth 3D pose annotation. Using this labeled data, a supervised learning algorithm, often a deep neural network, is trained to learn the correlation between the input data and the corresponding 3D poses. The training process involves tweaking the model’s parameters to minimize the difference between the predicted 3D poses and the ground truth 3D poses. This is accomplished by defining an appropriate loss function, such as mean squared error (MSE) or L1 loss.	[46,47,48,49]
Semi-Supervised Learning	Refers to an algorithm that conducts supervised learning when only a subset of the input data is labeled. The algorithm utilizes both labeled and unlabeled data for training the model. The model is initially trained on the labeled data, whereas the unlabeled data are employed to regulate the learning process or enhance generalization.	[50,51,52]
Weakly Supervised Learning	These methods do not use exact 3D pose annotations; rather, they utilize less precise data like 2D joint locations or multi-view images. The model could be trained using these 2D joint annotations when direct 3D pose labels are not available. Consequently, the model learns to estimate the 3D human pose from these 2D joint locations without any direct supervision related to the 3D poses themselves.	[53,54,55,56,57,58]
Unsupervised learning	These algorithms learn from input variables without having any associated output variables. In the context of 3D human pose estimation, it does not utilize any 3D data or additional views. The objective of unsupervised learning is to deduce the 3D pose structure directly from unlabeled 2D data, without the necessity for explicit 3D pose annotations. Some methods employ strategies like structure-from-motion or multi-view geometry, using multiple 2D views to infer relative 3D poses. Alternatively, some methods use models such as autoencoders or generative adversarial networks (GANs) to learn a latent representation of 3D poses.	[59,60,61,62,63]
Self-Supervised Learning	This is a specific type of unsupervised learning that makes use of the inherent structure or information within the data to generate its own labels. The model produces its own training labels using the available 2D annotations or certain substitute tasks. Additionally, some self-supervised methods may employ the concept of temporal consistency.	[64,65,66,67,68,69,70,71,72]

**Table 2:** Overview of acronyms and technical terms.

Acronym	Meaning	Explanation
2D	Two-dimensional	Refers to something having width and height but no depth. In the context of pose estimation, 2D refers to poses estimated within a two-dimensional space, such as an image.
3D	Three-dimensional	Refers to something having width, height, and depth. In the context of pose estimation, 3D refers to poses estimated within a three-dimensional space, providing a more realistic representation of human poses.
PCA	Principal component analysis	A statistical procedure that uses an orthogonal transformation to convert a set of observations of possibly correlated variables into a set of values of linearly uncorrelated variables called principal components.
CNN	Convolutional neural network	A type of artificial neural network used in image recognition and processing that is specifically designed to process pixel data.
LSTM	Long short-term memory	These models utilize long short-term memory units to capture temporal information across video frames.
TCN	Temporal convolutional network	These networks are designed for sequence modeling and use dilated convolutions and causal connections to capture long-range dependencies in time-series data.
GCN	Graph convolutional network	These models operate directly on graphs and can take into account the structure of the graph and the attributes of its nodes and edges. They are employed for their ability to model non-Euclidean data structures like human skeletons.
Transformers	Transformer-based methods	These deep learning architectures utilize self-attention mechanisms to handle sequential data, including text or images. Transformers can discern long-range dependencies and spatial relationships among features and are capable of modeling global dependencies among all elements in a sequence.
SMPL	Skinned multi-person linear model	A method for estimating human body shape and pose from images.
MDNs	Mixture density networks	A type of neural network that can model a conditional probability distribution over a multi-modal output space.
IKNet-body	Inverse kinematics network	A type of network used to calculate the angles of joints in a mechanism (like a robotic arm or a human skeleton) to achieve a desired pose.
IEF	Iterative error feedback	A method used in machine learning to iteratively correct the errors made by a model.
P2P-MeshNet	Point-to-pose mesh fitting network	Incorporates a collaborative approach between a deep learning network, an inverse kinematics network (IKNet-body), and an iterative error feedback network (IEF), enabling improved accuracy in estimating 3D poses.
HOGs	Histogram of oriented gradients	A feature descriptor used in computer vision and image processing for the purpose of object detection.
LCN	Locally connected network	A type of neural network where each neuron is connected to its neighboring neurons, but not necessarily to all other neurons in the network.

**Table 3:** Comparative results of root-relative and absolute 3D multi-person pose estimation on MuPoTS-3D dataset.

	Method	3D-PCK	3D-PCK${}_{\mathrm{abs}}$
Top-down approaches	GnTCN [146]	87.50	45.7
	HDNet [143]	83.70	35.2
	HMOR [145]	82.00	43.8
	3DMPPE-POSENET [141]	81.80	31.5
Bottom-up approaches	Single shot [151]	72.7	20.9
	SMAP [149]	80.50	38.7
	Mutual Adaptive Reasoning [150]	81.5	39.5
Fusion approaches	TDBU_Net [51]	89.60	48.0
	Dual networks [52]	89.6	48.1

**Table 4:** Comparative analysis of networks for video-based human pose estimation.

Category	Performance	Complexity	Key Features	Best Used For
LSTM-based methods	Good with short-term patterns, struggle with long-term patterns	Low complexity	Work well with data that follow a sequence	Tasks with short-term sequential data
TCN-based methods	Great with long-term patterns	Low complexity	Use special connections to capture long-term patterns	Tasks needing to capture long-term patterns
GCN-based methods	Very accurate with network-like structures	High complexity	Capture relationships between different points, good for spatial relationships	Tasks where estimation involves a network-like structure
Transformers	Promising results with long-term patterns and hidden points	High complexity	Use attention mechanism, good for understanding spatial relationships	Tasks needing to understand long-term patterns and spatial relationships
Unified frameworks	High accuracy; performance depends on the mix of methods	High complexity	Combine advantages of different methods, needs careful setup	Complex tasks where combining different methods could improve results

**Table 5:** Quantitative comparison of state-of-the-art methods leveraging temporal information for 3D human pose estimation on Human3.6M under protocol 1, using ground truth 2D poses as input.

Method	Temporal Network Type	Frames Needed	Average MPJPE (mm)
Mixtse [167]	Transformer	243	21.6
U-CondDGConv [173]	GCN	243	22.7
GAST-Net [155]	GCN+TCN	243	25.1
StridedTransformer [166]	Transformer	243	28.5
P-STMO [70]	Transformer	243	29.3
Modulated GCN [119]	GCN	50	30.06
MHFormer [124]	Transformer	243	30.5
PoseFormer [168]	Transformer	81	31.3
Anatomy3D [162]	TCN	243	32.3
SemGCN [46]	GCN	50	33.53
Attention-based framework [174]	TCN	243	34.7
VideoPose3D [156]	TCN	243	37.2
Propagating LSTM [157]	LSTM	3	38.4

**Table 6:** Common databases for 3D human pose estimation.

	Dataset	Description	Evaluation Metrics
Single Person	HumanEva-I [83] 2010	Seven calibrated video sequences using multiple RGB and gray-scale cameras, synchronized with 3D body poses obtained using marker-based motion capture system. The database contains 4 subjects performing 6 common actions.	3D error metric
	Human3.6M [13] 2013	The most popular and biggest benchmark for 3D human pose estimation. 3.6 million indoor video frames and corresponding poses of 11 professional actors captured by MoCap system from 4 camera viewpoints. Subjects 9 and 11 are used for testing, as in prior studies.	MPJPE Procrustes aligned MPJPE MRPE
	MPI-INF-3DHP [116] 2017	It consists of more than 1.3 million frame captured with markerless motion capture using 14 RGB cameras, consisting of both constrained indoor and complex outdoor scenes. It has 8 subjects performing 8 activity sets.	MPJPE 3D_PCK AUC${}_{rel}$
Multiple Person	MuCo-3DHP [137] 2018	Training dataset which merges randomly sampled 3D poses from single-person 3D human pose dataset MPI-INF-3DHP to form realistic multi-person scenes.	MPJPE 3D-PCK AUC${}_{rel}$ 3DPCK${}_{abs}$
	MuPoTS-3D [137] 2018	A dataset used for testing real-world shots of a 3D human pose dataset containing 20 videos (8000 frames) captured in both indoor and outdoor scenes, with challenging occlusions and person–person interactions.	3D-PCK AUC${}_{rel}$ 3DPCK${}_{abs}$
	Muco-Temp [182] 2020	A dataset generated in the same way as MuCo-3DHP. It consists of videos instead of frames Usually used for temporal networks training.	3D-PCK AUC${}_{rel}$ 3DPCK${}_{abs}$ MPJPE MRPE

**Table 7:** Evaluation metrics.

	Evaluation Metric	Name	Description
Person-centric (relative pose)	3D error metric	3D error metric	Measures the average squared distance between the predicted pose coordinates and the actual ones.
	MPJPE	Mean per-joint position error	Is the mean Euclidean error averaged over all joints and all poses, calculated after aligning the human root of the estimated and ground truth 3D poses. $MPJPE=\frac{1}{T}\frac{1}{N}\sum_{t=1}^{T}\sum_{i=1}^{N}{∥({J_{i}^{\left(t\right)}-{J_{root}}^{\left(t\right)})-({\hat{J}}_{i}}^{\left(t\right)}-{{\hat{J}}_{root}}^{\left(t\right)})∥}_{2}$ The first protocol **(P1)** uses five subjects for training and two for testing, while the second protocol **(P2)** uses six subjects for training and one for testing. The third protocol **(P3)** splits the dataset in the same way as P1 but evaluates only sequences captured by the frontal camera in trial 1 without sub-sampling the original video. The error is averaged over 14 joints in P1 and P2 and a subset of 14 joints in P3. All protocols use Procrustes analysis to calculate the pose error.
	3DPCK	3D Percentage of correct keypoints	Measures the percentage of correctly estimated keypoints within a certain distance threshold. In studies, an estimated joint is considered correct if it is within a 150 mm distance from the corresponding ground truth joint.
	AUC${}_{rel}$	Area under 3D-PCK curve	This performance metric is calculated by plotting the PCK values against different distance thresholds and integrating the area under the curve. A higher value of this metric indicates better performance of the algorithm.
Camera-centric (absolute pose)	MRPE	Mean root position error	The average error in the absolute root joint (the hip) localization. $MRPE=\frac{1}{T}\sum_{t=1}^{T}{∥({J_{root}}^{\left(t\right)}-{{\hat{J}}_{root}}^{\left(t\right)})∥}_{2}$
	AP${}_{25}^{root}$	Average precision of the root	Allows the 3D human root location prediction error to be measured, which considers the prediction as correct when the Euclidean distance between the estimated and the ground truth coordinates is smaller than 25 cm.
	3DPCK${}_{abs}$	3D percentage of correct absolute keypoints	3DPCK without root alignment to evaluate the absolute poses. In studies, the threshold distance used for an absolute joint to be estimated as correct is 250 mm.

Note that *T* denotes the total number of test samples and *N* denotes the number of joints. Ground truth joint and the predicted joint are indicated by *J* and $\hat{J}$, respectively. *i* represents each joint from all joints and root represents the root-joint.

## Data Availability

This article is a comprehensive review and does not contain any new data.
